# Vitamin/mineral/micronutrient supplement for autism spectrum disorders: a research survey

**DOI:** 10.1186/s12887-022-03628-0

**Published:** 2022-10-13

**Authors:** James B. Adams, Jasmine Kirby, Tapan Audhya, Paul Whiteley, Jaclyn Bain

**Affiliations:** 1 Arizona State University, Tempe, USA; 2 New York University, New York City, USA; 3 ESPA Research, Sunderland, UK; 4 Southwest College of Naturopathic Medicine, Tempe, USA

**Keywords:** Autism, Autism spectrum disorder (ASD), Vitamins, Minerals, Micronutrients

## Abstract

**Background:**

Vitamin and mineral supplements are widely used by children and adults diagnosed with autism spectrum disorder (ASD). Several studies have reported benefits of such supplements in resolving nutritional deficiencies, treating various metabolic problems and improving symptoms and overall quality of life.

**Methods:**

This research survey collected evaluations from 161 people about the effectiveness of ANRC-Essentials Plus (ANRC-EP), a vitamin/mineral/micronutrient supplement designed for children and adults with autism. Although this was an open-label survey, results were compared with a three-month randomized double-blind placebo-controlled study of an earlier version of the supplement. Evaluations included the Parent Global Impressions of Autism (PGIA) and the Overall Benefit/Adverse Effect scale of the National Survey on Treatment Effectiveness for Autism (NSTEA).

**Results:**

The participants reported substantially higher Average PGIA Scores than the placebo group in a similar previous study, with an estimated effect size of 0.66. Based on the NSTEA questionnaire, 73% of participants rated the Overall Benefit as Moderate, Good, or Great, with scores that were substantially higher than the NSTEA study found for multi-vitamins, the average of 58 nutraceuticals, and the average of 28 psychiatric and seizure medications. The Overall Adverse Effect score was low (0.25/3.0), similar or slightly higher than other nutraceuticals, and much lower than the average of 28 psychiatric and seizure medications (0.9/3.0). Sub-analysis found that the Overall Benefit of ANRC-EP was not significantly affected by gender, age, autism severity, diet quality, self-limited diet, use of psychiatric or seizure medications, dosage, developmental history, intellectual disability, or seizures. This indicates that ANRC-EP may be beneficial for a wide range of children and adults with ASD.

A limitation of this study is the retrospective nature of the survey, and that participants who had good benefits were more likely to respond.

**Conclusions:**

This study found that ANRC-EP had significant benefits for a wide range of symptoms, and low adverse effects.

## Background

Vitamins and minerals are, by definition, substances which have been proven to be essential to human life, and which must be consumed in the diet to prevent nutritional deficiencies and related illnesses. In the United States, data from the National Health and Nutrition Examination Survey (NHANES) 2007–2010 (*n* = 16,444) found that many people consume less than the estimated average requirement (EAR) of many nutrients, including vitamin D (74%), vitamin E (67%), magnesium (46%), calcium (39%), and vitamin A (35%) [1]. Children and adults with autism spectrum disorder (ASD) may be particularly susceptible to nutritional insufficiencies and deficiencies as a consequence of often self-limited diets restricting themselves to a limited number of foods [2]. Such behaviors follow on from the presence of restricted and/or repetitive behaviors being a core diagnostic feature of ASD, influenced also by various sensory issues [3].

Children with ASD may have an increased need for vitamin/mineral supplementation, due to a variety of metabolic problems, including increased oxidative stress [4, 5], methylation pathway insufficiency [6, 7], mitochondrial disorders [8], cerebral folate transporter antibodies [9], sulfate deficiency [2] and lithium deficiency [2]. Vitamins and minerals have many roles, including serving as co-factors for many enzymatic reactions, and some individuals need higher levels of those co-factors due to individual variation in enzyme function (Ames 2002) and other genetically-controlled processes. It is estimated, for example, that approximately one-third of mutations in a gene result in its corresponding enzyme having a decreased binding affinity for co-factors resulting in a lower enzyme activity. About 50 human genetic diseases can be treated to some extent with administration of high doses of a vitamin co-factor, resulting in improved enzyme activity [10].

Dietary supplements are commonly used in autism, with one study reporting 54% of children with ASD taking dietary supplements, primarily multi-vitamin/mineral supplements [11]. That study also reported that although intake of several vitamins and minerals from diet alone was insufficient, the supplements used often failed to treat some deficiencies while providing excessive amounts of other nutrients.

Various vitamin/mineral/micronutrient supplements for people with autism have been investigated in multiple clinical trials. These include folinic acid/vitamin B12 [6], vitamin B6 [12], vitamin C [13], vitamin D [14], zinc [15], and iron [16], with most of them demonstrating some positive benefit in correcting insufficiency and/or improving symptoms [17]. Several other studies have highlighted how pregnancy levels of micronutrients may also influence the risk of offspring developing autism. Low levels of folic acid during pregnancy may be a risk factor for autism [18], and low or excessive levels of folic acid and B12 may increase the risk [19, 20]. Cumulatively, such data suggest that a carefully designed vitamin/mineral supplement may therefore be beneficial for people with autism.

Most relevant to this paper are three studies reporting on use of a multi-vitamin/mineral/micronutrient supplement for children with autism. One small randomized study found significantly greater improvement in gastrointestinal (GI) and sleep symptoms in a treatment group compared to placebo [21]. One larger randomized study [22], referred to in this paper as Adams 2011, began with an extensive analysis of nutritional and metabolic status and found many abnormalities in the ASD group compared to controls [2]. At baseline, regression analysis found that ASD symptom severity (evaluated on three different scales) was significantly associated with levels of several vitamins (adjusted R2 of 0.25–0.57) and minerals (adj. R2 of 0.22–0.38). After treatment, the study found significantly greater improvements in the treatment group compared to the placebo group on the Parent Global Impressions Scale (PGIA), and on its subscores of hyperactivity, tantrums, and overall symptoms. The results of that study were used to design a slightly different vitamin/mineral formulation, which was used in a randomized 12-month study in combination with 5 other nutritional/dietary treatments [23], referred to in this paper as Adams 2018. Most of the benefit occurred during the first three months, when the vitamin/mineral supplement and essential fatty acids were the primary interventions. The treatment group had significantly greater improvement than the control group on many different measures of autism, autism-related symptoms, non-verbal IQ, and overall developmental age. Parents rated the vitamin/mineral supplement and the essential fatty acids as the highest-rated treatments, with over 85% of participants stating a desire to continue treatment with them.

Based on those positive studies, an improved version of the vitamin/mineral/micronutrient supplement was marketed in 2015 by the non-profit, Autism Nutrition Research Center (ANRC), under the name ANRC Essentials. Following the Adams 2018 study, the preparation was reformulated and re-marketed as ANRC Essentials Plus (ANRC-EP). The focus of this paper is to report the results of a research survey of ANRC-EP consumers, to evaluate safety, efficacy, and factors which affect efficacy.

## Methods

### Study design

A retrospective survey of consumers who purchased ANRC-EP for a child or adult with ASD**.**

### Research survey

An email invitation to participate in a research survey was sent to all consumers who had purchased ANRC-EP online from ANRC in the 12 months prior to spring 2020. The email invited them to complete a ten-minute survey about the safety and efficacy of ANRC-EP, in return for a 50% discount on their next purchase.

The inclusion criteria used were a formal diagnosis of an autism spectrum disorder (self-reported by the participant) and use of ANRC-EP for 3 months or longer. Those with suspected autism but no formal diagnosis were not included in the study. There were no additional exclusion criteria. All methods were performed in accordance with the Declaration of Helsinki, and the study was approved by the Institutional Review Board of Arizona State University (STUDY00013167). Informed consent was obtained from all participants or their legal guardian.

The survey asked several demographic questions (see Table 1) as well as questions about dosing, perceived benefits, and adverse effects. Participants then completed the Parent Global Impressions of Autism (PGIA). The PGIA includes 20 questions about the effect of the treatment (ANRC-EP) on autism-related symptoms based on a 7-point scale. Participants were allowed to skip any question they did not wish to answer.

**Table 1:** Formulation of ANRC-EP (present study) and similar formulations used in Adams 2018 and Adams 2011 studies. The dosages are listed for 6 capsules, which is the daily dose for a 60 pound child; dosages are adjusted by bodyweight (1 capsule per 10 pounds bodyweight, to a maximum of 12 capsules for 120 pound or higher). Lower, gradually-increasing doses were recommended for the first month

	**ANRC Essentials Plus (current study)**	**2018 study**	**2011 study**
**Vitamins**			
Vitamin A (Retinyl palmitate)	2000 IU (600 mcg RAE)	975 IU	1000 IU
Carotenoids	6000 IU vitamin A as mixed carotenoids	5525 IU as beta carotene	3.6 mg mixed carotenoids
Vitamin C (Calcium ascorbate)	300 mg	500 mg	600 mg
Vitamin D (D3)	2500 IU (62 mcg)	1000 IU	300 IU
Vitamin E (alpha tocopherol)	67 IU ( 45 mg RAE)	150 IU	150 IU
Mixed Tocopherols	55 mg of mixed tocopherols		
Vitamin K	300 mcg	55 mcg (K1 and K2)	none
	200 mcg K1, 100 mcg K2 (as MK7)		
Vitamin B1 Thiamin (as thiamin hydrochloride)	30 mg	20 mg	20 mg
Vitamin B2 Riboflavin	40 mg	40 mg	20 mg
Vitamin B3 Niacin	20 mg Niacin (nicotinic acid)	25 mg (as inositol hexanicotinate)	15 mg niacin
	20 mg Niacinamide	10 mg Niacinamide	10 mg Niacinamide
	10 mg Nicotinamide Adenine Dinucleotide (NADH)	None	none
	10 mg nicotinamide riboside	none	none
Vitamin B5 Pantothenic Acid (as calcium d-pantothenate)	30 mg	30 mg	15 mg
Vitamin B6	40 mg	40 mg	40 mg
	20 mg pyridoxine hydrochloride, 20 mg pyridoxal 5 phosphate (P5P)	20 mg pyridoxine hydrochloride, 20 mg pyridoxal 5 phosphate (P5P)	pyridoxine HCL
Vitamin B12	600 mcg	500 mcg	500 mcg
	500 mcg as hydroxocobalamin and 100 mcg methylcobalamin	250 mcg as methylcobalamin and 250 mcg as cyanocobalamin	cyanocobalamin
Folate	600 mcg	600 mcg	550 mcg
	MTHF (methyl-tetra-hydrofolate)	(as folic acid, folinic acid, & L-5-methyltetrahydrofolate)	folinic acid
Biotin	500 mcg	225 mcg	150 mcg
**Minerals**			
Calcium	200 mg	70 mg	100 mg
	(as calcium ascorbate, calcium pantothenate, di-calcium malate)	(as calcium ascorbate)	(as calcium ascorbate)
Chromium	70 mcg	70 mcg	70 mcg
	(as chromium picolinate)	(as chromium amino acid chelate)	(as chromium amino acid chelate)
Iodine	100 mcg	100 mcg	100 mcg
	(as potassium iodide)		
Magnesium	200 mg	100 mg	100 mg
	(as Mg Citrate (75 mg elemental Mg), Mg Taurate (50 mg elemental Mg), Di-Magnesium Malate (75 mg elemental Mg)	as magnesium citrate	(as magnesium chloride hexahydrate)
Manganese	0.5 mg	1 mg	3 mg
	(as manganese aspartate)	(as manganese amino acid chelate)	(as manganese amino acid chelate)
Molybdenum	100 mcg	100 mcg	150 mcg
	(as molybdenum glycinate)	(sodium molybdate dihydrate)	(sodium molybdate dihydrate)
Potassium (as potassium chloride)	50 mg	50 mg	50 mg
Selenium (80% as selenomethionine, 20% as sodium selenite)	50 mcg	40 mcg	22 mcg
Zinc	15 mg	15 mg	12 mg
	(50% as zinc sulfate, 50% as amino acid chelate)	(as zinc gluconate)	(as zinc gluconate)
**Other Nutrients**			
Choline	250 mg	250 mg	250 mg
	(as choline bitartrate)	(as choline bitartrate)	choline chloride
Co-Enzyme Q10 (ubiquinone)	100 mg	50 mg	50 mg
Inositol	100 mg	100 mg	100 mg
Carnitine	300 mg	200	none
	l-carnitine	acetyl-l-carnitine	
Lithium (as lithium orotate)	350 mcg	350 mcg	500 mcg
Methylsulfonylmethane (MSM)	500 mg	500 mg	500 mg
N-acetyl-cysteine	100 mg	45 mg	50 mg
Taurine (as Mg taurate)	514 mg	None	none
Vanadium	none	25 mcg	none
Boron	none	250 mcg	none

This study design (survey of current/recent users) was chosen for several reasons. First, the short length of the survey encouraged high participation, resulting in good statistical power which allowed many sub-analyses to be conducted. Second, although the open-label nature of the participants results in some placebo-effect, this does not affect most of the sub-analyses, such as analysis of effect of age or gender, since the placebo-effect is likely to be similar for everyone. Third, the survey design allows evaluation of long-term effects of supplementation – some ANRC-EP consumers had been taking it for up to 6 years. Thus, this survey design of real-world use complements the previous randomized controlled studies [21–23]. Limitations of the study design are described in a section at the end of this paper.

The effect of ANRC-EP on symptoms was evaluated in several ways. The effect of ANRC-EP on autism-related symptoms was evaluated on the Parent Global Impressions of Autism. This is a slightly modified version of the PGI-II [23] which included 18 questions on ASD symptoms; two questions on “seizures” and “self-limited diet” were added for this study. The change in each of 20 symptoms is rated on a 7-point scale (-3: much worse; -2 worse; -1 slightly worse; 0 no change; + 1 slightly better, + 2 better, + 3 much better). Importantly, if a person does not present with a specific symptom, then a “not applicable” option can be chosen. This is important so that change in rare symptoms, such as seizures, are only evaluated in participants with those symptoms. An Average score is calculated by averaging all of the scores.

Participants were asked to provide an Overall Benefit score, on a scale of 0–4, and an Overall Adverse Effect score, on a scale of 0–3, using the same scale as in the National Survey on Treatment Effectiveness for Autism (NSTEA) [24, 25]. If adverse effects occurred, participants were asked to indicate which ones based on a list from the NSTEA. This allows a direct comparison of the results of this study with that study.

Diet was assessed with two questions, one about the overall quality of the diet, and one about the variety of foods eaten by the participant.

### Formulation

The formulation of ANRC-EP is listed in Table 1, and compared against the formulations used in the Adams 2018 and Adams 2011 studies. Consumers were provided with a dosage sheet based on bodyweight, and given directions on how to slowly increased the dosage during the first month, and to stop at a lower dosage if adverse effects were observed.

### Statistical analysis

The primary methods for statistical analysis were calculations of averages, t-test comparisons of different groups/effects, and correlation analysis. Results with *p*-values below 0.05 were deemed statistically significant in this exploratory study. However, it should be noted that no control for multiple analyses were conducted, because the different evaluations were considered to be largely independent of one-another. This is a limitation of this study.

Raw effect sizes were calculated as the degree of change in the PGIA divided by the standard deviation (no control for placebo effect); these provide an upper bound on the possible effect size. Net effect sizes (Cohen’s d) were estimated as the difference between the PGIA score in the present study compared to the placebo group in the 2011 study, divided by the difference in the average of their standard deviations; since this is a comparison between two similar but different studies we call this an “estimated” net effect size.

## Results

The survey was completed by 233 people. Of those responses, 22 were not included because they had taken ANRC-EP for less than 3 months. Also, 11 had a diagnosis of attention-deficit hyperactivity disorder (ADHD) only, 15 had some ASD symptoms but did not have a formal diagnosis at the time of survey, and 24 had other diagnoses (not ASD); these groups were not included in the analysis. The remaining 161 participants had a diagnosis of Autism or Autism Spectrum Disorder (ASD), and were included in the analysis. The demographics of the study are listed in Table 2. The duration of usage of ANRC-EP ranged from 3 months to over 4 years, with the median usage of approximately 12 months.

**Table 2:** Demographics of Autism and ASD Participants

**Gender**	
Male	138 (86%)
Female	23 (14%)
**Age**	12.7 ± 9.1 years (1–74 years)
**Age Distribution**	
1–5 years	27 (17%)
5–10 years	53 (33%)
11–15 years	32 (20%)
16–20 years	31 (19%)
21–25 years	6 (4%)
26–30 years	4 (2%)
31 + years	7 (4%)
**Person completing the questionnaire**	
Parent/Guardian	153 (95%)
Grandparent	2 (1%)
Self	6 (4%)
**Diagnosis**	
Autism	84 (52%)
ASD	77 (48%)
**Country of Residence**	
United States	153 (95%)
Non-US	8 (5%)
**Race**	
White/Caucasian	103 (64%)
African American/Black	6 (4%)
Asian Indian	14 (9%)
Asian non-Indian	17 (11%)
Other	21 (13%)
**Ethnicity**	
Hispanic	17 (11%)
Non-Hispanic	141(88%)
unspecified	3 (2%)
**Autism severity**	
Mild	39 (24%)
Moderate	76 (47%)
Severe	45 (28%)
**Developmental History of Autism**	
Early onset	82 (51%)
Normal development, followed by regression or plateau	79 (49%)
Age at regression/plateau	20 ± 11 months
**Seizures**	10 (6%)
**Intellectual Disability**	31 (19%)
**Asthma**	7 (4%)
**Use of Psychiatric or Seizure Medications**	65 (39%)
**Duration of usage of ANRC-EP**	
3–5 months	47
6–8 months	15
9–12 months	21
1–2 years	35
2–3 years	24
4 + years	19

Table 3 describes the diet quality of participants, which ranged from poor to excellent, and 52% of participants reported a somewhat or very limited variety of foods consumed in the diet.

**Table 3:** Diet Quality and Limited Diet

**Diet Quality**	
Excellent: plenty of vegetables, fruit, and protein; minimal junk food	56 (35%)
Very good: good amount of vegetables, fruit, and protein; modest amount of junk food	32 (20%)
Good: some vegetables, fruit, and protein; some junk food	26 (16%)
Fair: somewhat limited amount of vegetables, fruit, or protein; substantial amount of junk food	28 (17%)
Poor: limited amounts of vegetables, fruit, or protein; high amounts of junk food	19 (12%)
**Does the participant eat a limited variety of foods?**	
No	40 (25%)
Slightly limited	37 (23%)
Somewhat limited	61 (38%)
Very limited	23 (14%)

Table 4 provides information on the dosage of ANRC-EP. Most participants (81%) reported slowly increasing dosage over 1 month per ANRC guidelines, which were designed to reduce the risk of adverse effects (AE). Most participants reported consuming the full dose, but 36% consumed a lower dose, due to adverse effects at higher dosages (15%) or other non-AE reasons (21%).

**Table 4:** Dosage Information for ANRC Essentials Plus (ANRC-EP)

**Dosage Frequency**	
Daily	151 (94%)
Less than daily (average 3.4 days/week)	10 (6%)
**Dosage form**	
Powder	66 (41%)
Capsule	95 (59%)
**Was the dosage slowly increased during the first month per ANRC guidelines?**	
Yes	131 (81%)
No	29 (19%)
**Average Dose as a fraction of Recommended Dose (based on reported pill/powder count)**	
Capsule	56%
Powder	62%
**Are you using less than the recommended full dose due to possible adverse effects?**	
Full Dose	103 (64%)
Less than full dose	58 (36%)
**Reasons for less than full dose**	
*Adverse effect (AE) reasons for taking lower dose*	
Hyperactivity at higher dose	11 (7%)
Anxiety/aggression/irritability at higher dose	5 (3%)
Reflux at higher dose	2 (1%)
Other AE at higher dose	6 (4%)
*Non-adverse-effect reasons for taking lower dose*	
Cost	7 (5%)
Scheduling problems	6 (4%)
Compliance due to taste/number of pills	5 (3%)
Other (not AE)	13 (8%)
**When the participant started ANRC Essentials Plus, did they make any other changes in medications, supplements, diets, or treatments?**	
No other changes	48%
Some other changes, but I am fairly confident that the changes I reported are due to ANRC Essentials Plus	29%
Some other changes, so I am unsure if the changes I reported were due to ANRC Essentials Plus or other medications/supplements/diets/treatments	23%

Table 5 reports on the change in symptoms based on the PGIA questionnaire, and compares them to the results of the treatment and placebo groups in the Adams 2011 study. The scores for the 2011 Treatment study are also listed in Table 5 for comparison; that study used an earlier version of the PGIA, with fewer questions. For the questions which were common between the two studies, the average symptom improvements were very similar (0.82 vs 0.70, n.s.), with a very high correlation (*R* = 0.87) for comparison of the set of PGIA scores between the two groups; i.e., both studies agreed closely as to which symptoms improved least/most. The present study found a substantially higher Average score than the placebo group in the 2011 study (0.82 vs. 0.37, *p* < 0.001), and significantly higher scores in most symptoms including cognition/thinking, receptive language/communication, play/leisure skills, eye contact, tantrums/meltdowns, sleep, hyperactivity, and overall symptoms, and trends towards greater improvement in expressive language/speech and stools/GI problems. So, although caution is needed in comparing different study designs, it appears that the present results are very similar to those of the treatment group in a randomized, double-blind, placebo-controlled study, and substantially higher than the placebo group in that study.

**Table 5:** Change in Symptoms according to the Parent Global Impressions of Autism Score, for the present study, sorted in order from most to least improved, followed by the Overall rating and Average rating. Results for a previous study (treatment and placebo groups), are shown for comparison. The rating is done on a 7-point scale, from -3 (much worse) to 0 (no change) to + 3 (much better). A t-test comparing the present study vs. the treatment group and vs the placebo group of the Adams 2011 study are shown. The raw effect size and net effect size (Cohen’s d) are shown

Symptom	Present Study (ANRC-EP)	2011 Treatment Study (treatment group)	2011 Treatment Study (Placebo group)	ttest (present vs 2011 treatment	Ttest (present vs 2011 placebo	Raw Effect size (present study)	Estimate of Net Effect Size (Cohen’s d)
Receptive Language/Comprehension	1.06 (1.0)	0.91 (1.1)	0.51 (0.92)	n.s	*p* < 0.001	1.04	0.57
Cognition/Thinking	1.05 (1.0)	0.74 (1.0)	0.43 (0.84)	n.s	*p* < 0.001	1.04	0.64
Attention/Focus	1.04 (1.0)					1.04	
Expressive Language/Speech	0.97 (1.0)	1.09 (1.1)	0.71 (0.92)	n.s	*p* = 0.10	0.95	0.26
Play/Leisure Skills	0.94	0.81 (0.88)	0.51 (0.87)	n.s	*p *= 0.005	0.95	0.45
Sociability	0.86 (0.94)	0.77 (0.95)	0.63 (0.92)	n.s	n.s	0.91	0.25
Eye Contact	0.76 (0.91)	0.66 (0.98)	0.40 (0.85)	n.s	*p* = 0.01	0.84	0.40
Irritability/Mood	0.74 (1.2)					0.63	
Tantrums/Meltdowns	0.73 (1.2)	0.4 (1.1)	-0.10 (0.80)	*p* = 0.07	*p* < 0.001	0.63	0.77
Stools/GI Problems	0.65 (1.0)	0.68 (1.1)	0.35 (1.1)	n.s	*p* = 0.10	0.62	0.28
Sensory Sensitivity	0.59 (0.95)					0.62	
Anxiety	0.57 (1.1)					0.52	
Sleep	0.52 (0.96)	0.3 (0.87)	0.13 (0.99)	n.s	*p* = 0.02	0.54	0.40
Self-limited Diet (will only eat a few foods)	0.48 (0.88)					0.55	
Self-Abusive	0.47 (1.0)					0.46	
Aggression	0.45 (1.1)					0.41	
Stimming/Perseveration	0.39 (1.1)					0.34	
Hyperactivity	0.31 (1.1)	0.31 (0.67)	-0.06 (0.55)	n.s	*p* = 0.003	0.28	
Seizures	0.19 (0.62)					0.32	
**OVERALL Autism/Related Symptoms**	1.11 (0.98)	1.02 (1.0)	0.56 (0.86)	n.s	*p* < 0.001	1.13	0.57
**Average of All Scores**	0.75 (0.77)					0.97	
**Average of scores used in 2011 study**	0.82 (0.76)	0.70 (0.65)	0.37 (0.54)	n.s	*p* < 0.001	1.07	0.66

Table 5 also lists the raw effect size (not counting placebo effect) and the estimated net effect size (using the placebo effect from the Adams 2011 study). Caution is needed in using the placebo effect from a different study to estimate the net effect size in this study. The estimated net effect sizes were “medium” (defined as Cohen’s d of 0.5–0.79) for the Average of all scores, Overall, tantrums/meltdowns, cognition/thinking, and receptive language, and “small” (defined as 0.2–0.49) for play/leisure skills, sleep, eye contact, stools/GI problems, expressive language/speech, and sociability). For the symptoms which were not evaluated in the 2011 study, several had raw effect sizes above 0.6, so they may also be possible areas of improvement, including attention/focus, irritability/mood, sensory sensitivity, and anxiety.

**Fig. 1:**
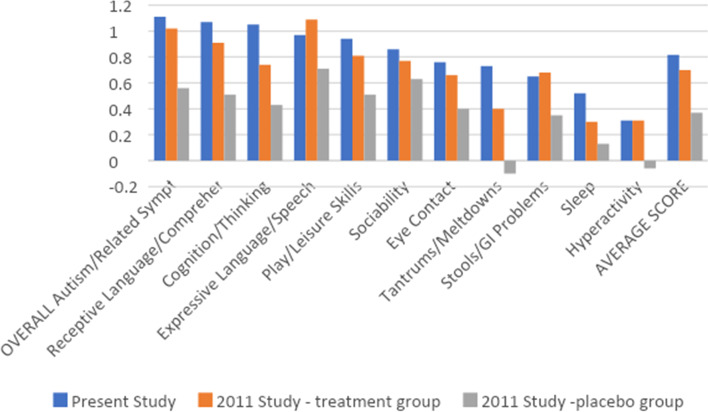
PGIA scores for the present study and the 2011 study (treatment and placebo groups), with symptoms sorted from highest to least improvement, followed by the Average Score of all symptoms. Only the symptoms scored in the 2011 version of the PGIA are shown

Figure 1 shows a plot comparing the results of the present study with the results of the Adams 2011 study (treatment group and placebo group), ordered from most to least benefit, followed by the Average Score. This shows that the present study had very similar results to the Adams 2011 treatment group, and substantially higher than for the placebo group.

**Fig. 2:**
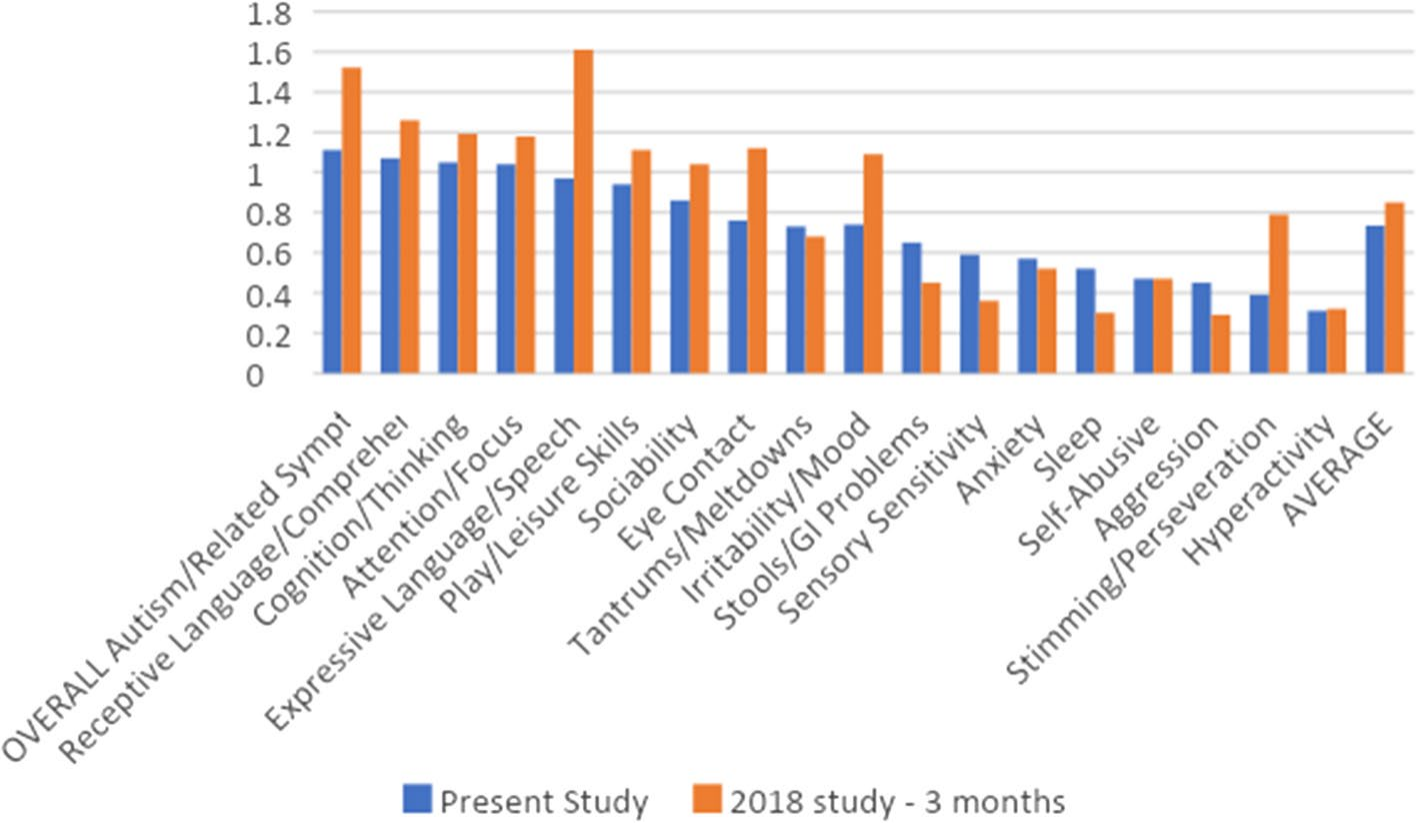
PGIA scores for the present study and the treatment arm of the 2018 study at 3 months, with symptoms sorted from highest to least improvement, followed by the Average score of all symptoms. Only the symptoms scored in the 2018 version of the PGIA are shown

Figure 2 shows a comparison of the PGIA scores for the present study compared to a comprehensive diet and nutrition study (Adams 2018) at 3 months of treatment, at which point participants had started the vitamin/mineral supplement at day 0, essential fatty acids (fish oil) at day 30, and Epsom salt baths at day 60, with evaluations at day 90. There was a very high correlation (*R* = 0.85) of the set of PGIA scores between the present study and the treatment group of the 2018 study at 3 months. The Average score of the 2018 study at 3 months is slightly higher than the present study (0.85 and 0.73, respectively), which may represent the additional effect of fish oil and Epsom salt baths, and suggests that most of the benefit from the comprehensive diet study was from the vitamin/mineral supplement.

Table 6 lists the rating of Overall Benefit for the present study based on the NSTEA scale (0 to 4) which was 2.32 ± 1.2, which is between “Moderate Benefit” and “Good Benefit.” The median time required to observe improvement was approximately 5–8 weeks, and 91% noticed significant improvement by 9–12 weeks. After the first few months, most experienced stable benefits, with 9% reporting some loss of benefit, and 21% reporting some increase in benefit.

**Table 6:** Rating of Overall Benefit of ANRC Essentials Plus (ANRC-EP), based on the NSTEA scale, and related information

**Overall Benefit** (NSTEA scale)		2.32 ± 1.2 (rated on a scale of 0–4)
No Benefit (0)		13 (8%)
Slight Benefit (1)		30 (19%)
Moderate Benefit (2)		47 (29%)
Good Benefit (3)		43 (27%)
Great Benefit (4)		27 (17%)
**How quickly did you observe significant change in symptoms?**		
1–2 weeks		18 (11%)
3–4 weeks		51 (32%)
5–8 weeks		39 (24%)
9–12 weeks		14 (9%)
13 weeks or longer		16 (10%)
Not applicable—no changes observed		23 (14%)
**How did benefits change after the first few months**		
Some loss of benefit		13 (9%)
Stable benefit		102 (70%)
Some increase of benefit after first few months		30 (21%)
**Did the participant ever stop taking ANRC Essentials Plus, and did you notice a loss of benefits?**		
Never stopped	92 (57%)	
Stopped, but no change	27 (17%)	
Stopped, and noticed some changes	42 (26%)	
Speed of change (9 responses)	Average 10 days (1 day to a few weeks)	
Worsening of symptoms when stopping ANRC^a^	38 responses Alertness/focus: 12 General symptoms: 6 Communication: 5 Stimming: 5 Cognition: 4 Behavior: 4 Agitation: 4 Mood: 4 Fatigue: 3 Anxiety: 3 Pica: 3 Hyperactivity: 2 Sleep: 2 Emotion Regulation: 2 Aggression: 2 GI: 2 Eye contact: 1 Depression: 1 Lost weight: 1 Sensory: 1 Sick more: 1 Eczema: 1 Night leg pain: 1	
Improvement of symptoms when stopping ANRC^a^	2 responses (they also reported worsening of symptoms listed above) body odor 1; hyperactivity/irritability: 1	

^a^These answers are based on analysis of free-form text, so in future a listing of actual symptoms might provide a more complete picture

Table 6 also lists the effects of stopping ANRC-EP. 57% of participants never stopped, 17% stopped with no changes, and 26% stopped and observed changes. Many respondents commented that they restarted the supplement after stopping it and observing a loss of benefits. There were 36 responses indicating worsening of symptoms when stopping the supplement, and 2 responses indicated both positive and negative changes.

Table 7 describes the overall level of adverse effects (AE), and the specific adverse effects that were reported using the NSTEA questionnaire. The Overall Adverse Effect score was 0.25 on a scale of 0–3 (0 = none, 1 = mild, 2 = moderate, 3 = severe). Most participants (79%) reported there were no adverse effects, and the reported AE’s were mostly mild. Overall, 78% of participants who experienced AE’s reported that reducing the dose helped reduce the adverse effects.

**Table 7:** Adverse Effects. The severity of Overall Adverse Effects based on the 0–3 scale of the NSTEA scale, followed by the rate of occurrence of individual symptoms, arranged in order from most to least common. The bottom section includes a list of possible symptoms which were asked about but which did not occur

**Overall Adverse Effects (NSTEA scale)**	0.25 ± 0.54 (on a scale of 0–3)	
No adverse effects (0)	127 (79%)	
Mild Adverse Effects (1)	27 (17%)	
Moderate Adverse Effects (2)	5 (3%)	
Severe Adverse Effects (3)	1 (0.6%)	
**Symptom**	**Percentage**	**N**
Hyperactivity	9%	14
Stimming/perseveration	6%	9
Irritability	4%	7
Aggression/agitation	4%	6
Behavior problems	4%	6
Gastrointestinal Problems	4%	6
Anxiety	3%	5
Sleep problems	2%	4
Bedwetting/bladder control	2%	3
General Worsening	1%	2
Cognition (ability to think)	1%	2
Tics/abnormal movements	1%	2
Dry mouth	1%	1
Headache/migraine	1%	1
Nausea	1%	1
Weight gain	1%	1
Other (not in original list, but reported by participant): Odd Body Odor	1%	1
**Symptoms which were asked about but were reported as not occurring**		
Depression	0%	0
Dizziness/unsteadiness	0%	0
Fatigue/drowsiness	0%	0
Liver/kidney problem	0%	0
Loss of appetite	0%	0
Rash	0%	0
Seizures	0%	0
Self-injury	0%	0
Weight loss	0%	0
**How long did adverse effects last?**	% of 32 respondents	
A few days	13%	4
1–2 weeks	13%	4
Several weeks or more	28%	7
Until reducing the dose	47%	15
Until totally stopping the supplement	6%	2
**Did reducing the dose help reduce the adverse effects?**	% of 32 respondents	
Yes	78%	25
No	22%	7

Table 8 lists the results of the analysis of the effect of many factors on Overall Benefit score (using the NSTEA scale of 0–4). The participants who followed the recommendation to gradually increase the dosage during the first month had significantly higher Overall Benefit (2.38 vs 1.70, *p* = 0.006). Also, there was a weak but significant positive correlation of longer usage of ANRC-EP with greater Overall Benefit (*R* = 0.22, *p* = 0.01). The other factors did not have a significant effect on Overall Benefit, including gender, age, autism severity, diet quality, limited variety of foods, use of psychiatric or seizure medications, dosage (full or reduced), developmental history (early onset of autism vs. typical development followed by regression and/or plateau), intellectual disability, or seizures.

**Table 8:** Effect of many factors on the Overall Benefit score (using the NSTEA scale of 0–4). Significant results are highlighted in bold

	**Overall Benefit (NSTEA scale of 0–4)**	**T-test result**	**Correlation**
**Gender**		n.s	
Male	2.27 ± 1.2		
Female	2.17 ± 1.2		
**Age**			*R* =—.0.01 (n.s.)
1–5 years	2.26		
6–10 years	2.10		
11–15 years	2.38		
16–20 years	2.58		
21–25 years	2.0		
25–30 years	2.50		
31 + years	1.63		
**Autism Severity**			*R* =—0.04 (n.s.)
Mild (1)	2.38		
Moderate (2)	2.20		
Severe (3)	2.24		
**Diet Quality**			*R* = 0.05 (n.s.)
Excellent (5)	2.41		
Very Good (4)	2.13		
Good (3)	2.04		
Fair (2)	2.32		
Poor (1)	2.21		
**Limited Variety of Foods**			*R* = 0.03 (n.s.)
Not limited	2.25		
Slightly limited	2.16		
Somewhat limited	2.28		
Very limited	2.35		
**Use of Psychiatric or Seizure Medications**		n.s	
Yes	2.14		
No	2.34		
**Dosage**		n.s	
Full dose	2.43		
Reduced dose	2.21		
**Was the dosage gradually increased during the first month per ANRC guidelines?**		***p* = 0.006**	
Yes	2.38		
No	1.70		
**Duration of ANRC-EP usage**			***R* = 0.22 (*p* = 0.01)**
3–5 months	1.9		
6–8 months	2.2		
9–12 months	2.2		
1–2 years	2.4		
2–3 years	2.6		
4 + years	2.7		
**Developmental History**		n.s	
Early Onset of autism	2.14		
Normal development, followed by regression or plateau	2.38		
**Intellectual Disability**		n.s	
Yes	1.97		
No	2.34		
**Seizures**		n.s	
Yes	2.00		
No	2.29		

Table 9 lists a comparison of the results of the present study with the National Survey on Treatment Effectiveness for Autism (NSTEA) [24, 25], using the NSTEA scale for rating Overall Benefits and Overall Adverse Effects. ANRC-EP had a higher Overall Benefit rating than the other treatments, and a low AE score.

**Table 9:** Overall Benefit and Overall Adverse Effect scores for ANRC-EP compared to the results of the NSTEA survey

	**Overall Benefit** **(0–4)**	**Overall Adverse Effect** **(0–3)**
**ANRC-EP (present study)**	2.32	0.25
**Average of general multi-vitamin/mineral supplements**	1.4	0
**Average of high-dose multi-vitamin/mineral supplements**	1.9	0.2
**Average of high dose multivitamin for autism**	1.8	0.2
**Average of 58 nutraceuticals**	1.59	0.1
**Average of 28 psychiatric and seizure medications**	1.39	0.9

## Discussion

Overall, 92% of participants reported positive benefit on the Overall Benefit score (based on the NSTEA scale), with 44% reporting “good” or “great” benefit following use of the ANRC Essentials Plus (ANRC-EP) (see Table 6). The degree of benefit was significantly higher in people who followed the ANRC guidelines of starting with a lower dose and then gradually increasing during the first month; presumably this is due to giving the body more time to adjust to the higher intake of nutrients. The degree of benefit was also slightly higher in people who took it for a longer period of time; this could however be partly due to cessation of use by people who had less benefit, but also the data in Table 6 suggests that some participants (21%) had some increase in benefit after the first several months. Interestingly, other factors such as age, gender, autism severity, and diet quality did not have a significant effect on degree of benefit, suggesting that the supplement may benefit a large fraction of individuals with autism/ASD.

This study also found that ANRC-EP had a good safety profile, with an Overall Adverse Effect rating of 0.25 ± 0.54 on a scale of 0–3 (see Table 7). Only 21% of participants reported one or more adverse effects (AE), which were mostly mild, and in 78% of cases could be reduced by reducing the dose.

The results of this study for the PGIA were very similar to that of the treatment group in a previous study using an earlier version of this supplement (Adams 2011), with a high correlation between studies as to which symptoms improved least/most. Comparing the results of the present study to the placebo group of the 2011 study, this study found a significantly higher improvement in the Average score (averaging over all 11 symptoms) and on 8 of 11 symptoms. This suggests that ANRC-EP provides a wide range of symptom improvements compared to placebo for individuals with ASD.

The current study also evaluated changes in 9 additional symptoms not evaluated in the Adams 2011 study, and found improvements in attention/focus (average improvement of 1.04), irritability/mood (0.74), sensory sensitivity (0.59), anxiety (0.57), self-limited diet (0.48), self-abusive (0.47), aggression (0.45), and stimming/perseveration (0.39). However, it seemed to have little effect on seizures (0.19) on the small number of participants with seizures (*n* = 11). The placebo effect for those symptoms is unknown, so a randomized placebo-controlled study is needed to confirm those possible improvements.

One major question is the reason why ANRC-EP seems to improve symptoms. The current study found no correlation of improvement with diet quality or self-limited diet. Therefore, it seems likely that the major reason for improvement is not due to poor diets, but rather due to nutritional and metabolic differences in people with autism that require high levels of supplementation. Vitamins and minerals are important co-factors for many enzymes, and some individuals may need higher levels of those co-factors due to individual variation in enzyme function [10]. The Adams 2011 study involved an extensive study of nutritional and metabolic status of children with ASD vs. controls, and found many very significant (*p* < 0.001) differences, including lower levels of biotin, plasma glutathione, RBC (red blood cell) SAMe (S-adenosylmethionine), plasma uridine, plasma ATP (adenosine triphosphate), RBC NADH (Nicotinamide Adenine Dinucleotide), RBC NADPH (nicotinamide adenine dinucleotide phosphate), plasma sulfate (free and total), and plasma tryptophan; and higher levels of oxidative stress markers and plasma glutamate. Treating those children with the vitamin/mineral supplement resulted in significant improvements in most of those biomarkers, and presumably similar effects occurred in this study.

Several of the vitamins, minerals, and micronutrients in ANRC-EP have individually been investigated and found to be beneficial in children and/or adults with ASD in randomized, double-blind, placebo-controlled studies. These include studies of Methyl-B12, folinic acid, and trimethylglycine (TMG) [6, 7, 26], high dose folinic acid [27, 28], high dose vitamin B6 with magnesium [29], vitamin D [30–32], high-dose carnitine [33, 34], CoQ10 [35], and high-dose Vitamin C [13, 36]. Similarly, Open-label trials of vitamin/mineral supplementation for ASD have reported benefits for vitamin A [37], iron [16] and zinc [15], in children with ASD, and a case study of high dose biotin [38].

In summary, several of the vitamins, minerals, and other micronutrients in ANRC-EP have been found to be individually beneficial in children and/or adults with ASD, thus explaining why combining them in ANRC-EP is helpful.

Figure 2 compares the results of the present study vs. the 2018 comprehensive diet and nutrition study at 3 months, at which point participants had started the vitamin/mineral supplement at day 0, essential fatty acids day 30, and Epsom salt baths day 60, with evaluations at day 90. The PGIA scores for the two studies were highly correlated, and the 2018 study had only slightly higher scores (16% higher), suggesting that most of the benefit was due to the vitamin/mineral supplement, with a small additional benefit due to the essential fatty acids and Epsom salt baths.

This study used the same rating scale for Overall Benefit and Overall Adverse Effect as used in the NSTEA studies [24, 25]. As shown in Table 9, ANRC-EP had a substantially higher Overall Benefit score than the multivitamins, suggesting that this was a better formulation for most children and adults with ASD. It also had a better Overall Benefit score than the average of 58 nutraceuticals and average of 28 psychiatric and seizure medications, suggesting that this may be one of the more effective medical treatments for autism. ANRC-EP had a low Overall Adverse Effect score (0.25/3.0), slightly higher than other multivitamins and nutraceuticals, but much lower than the average of 28 of the most commonly used psychiatric and seizure medications. Thus, this data suggests that ANRC-EP is both safe and effective, and worth considering for many children and adults with ASD.

## Strengths and limitations

### Strengths

This study involved a substantial number of participants (*n* = 161), allowing many analyses and sub-analyses to be conducted. The PGIA results of this study correlated highly (*R* = 0.87) with the results of the treatment group in the Adams 2011 randomized study, and the results of the 2018 study at 3 months (*R* = 0.85).

### Limitations

One limitation is that this study did not involve a placebo group. A similar study (Adams 2011) of an earlier version of this supplement did however include a placebo group, so it was possible to cautiously compare the present findings against the results of that study, although the treatment duration is different. Another limitation is that this was a retrospective survey predominantly relying on parental reports. That being said, this is also a strength of the study given the unique viewpoint offered by caregivers who can offer highly accurate accounts [39]. Likewise, the survey was only sent to consumers who purchased ANRC-EP within the last 12 months, and since many participants were still using ANRC-EP at the time of study, recall issues such as telescoping effects are likely to be minimal. Another issue is that there is probably some under-reporting of adverse effects, in that people who stopped using the supplement before 3 months were not included. However, in the Adams 2011 study, there were only 3 participants out of 72 in the treatment group (4%) who dropped out of the study due to adverse effects, which suggests that this bias is probably modest. Another limitation is that participants who observed benefit are more likely to stay on the supplement and hence respond to the survey, but the similarity in PGIA results to the Adams 2011 and Adams 2018 studies suggest this effect was small. Another limitation is that the time scale of this study (3 months to 5 years) is different than the time scale of the Adams 2011 study (3 months) and Adams 2018 study (we compared with the results at 3 months); however, since participants reported most changes had occurred by 3 months, this is probably a smaller effect. Some families reported starting ANRC-EP with other treatments, which limits the reliability of their results.

## Conclusions

This study found that ANRC-EP had very similar benefits (PGIA scores) compared to the treatment group in a previous randomized double-blind placebo-controlled study, and significantly higher scores than the placebo group of that study, including on the Average of all scores, and 8 of 11 subscores, and a trend towards improvement on 2 of the other subscores. The net effect size (Cohen’s d) was estimated to be 0.66 averaging over 11 symptoms on the PGIA, which is “medium effect” (defined as d = 0.50–0.79).

Using the NSTEA scale, 73% of participants rated the benefit as Moderate, Good, or Great, and the Overall Adverse score was low (0.25/3.0)).

The participants who followed the recommendation to gradually increase the dosage during the first month had significantly higher Overall Benefit. Most participants observed benefits within 3 months of usage, and 21% of participants reported additional benefits after longer-term use.

In summary, the studies to date of ANRC-EP and its earlier versions consistently found many positive benefits with few adverse effects for a wide range of children and adults with autism/ASD.

## Data Availability

The datasets used and/or analyzed during the current study are available from the corresponding author on reasonable request.

## Abbreviations

ADHD: Attention-deficit hyperactivity disorder
AE: Adverse Effects
ANRC: Autism Nutrition Research Center
ANRC-EP: Autism Nutrition Research Center Essentials Plus
ASD: Autism Spectrum Disorder
ATP: Adenosine triphosphate
EAR: Estimated average requirement
MSM: Methylsulfonylmethane
NADH: Nicotinamide Adenine Dinucleotide
NADPH: Nicotinamide adenine dinucleotide phosphate
NHANES: National Health and Nutrition Examination Survey
NSTEA: National Survey on Treatment Effectiveness for Autism
PGIA: Parent Global Impressions of Autism
SAMe: S-adenosylmethionine
